# Environmental control of the microfaunal community structure in tropical bromeliads

**DOI:** 10.1002/ece3.2797

**Published:** 2017-02-09

**Authors:** Pavel Kratina, Jana S. Petermann, Nicholas A. C. Marino, Andrew A. M. MacDonald, Diane S. Srivastava

**Affiliations:** ^1^ School of Biological and Chemical SciencesQueen Mary University of LondonLondonUK; ^2^ Biodiversity Research Centre and Department of ZoologyUniversity of British ColumbiaVancouverBCCanada; ^3^ Department of Ecology and EvolutionUniversity of SalzburgSalzburgAustria; ^4^ Programa de Pós‐Graduação em EcologiaDepartmento de EcologiaInstituto de BiologiaUniversidade Federal do Rio de Janeiro (UFRJ)Rio de JaneiroRJBrazil

**Keywords:** aquatic microfauna, community structure, environmental sorting, natural microcosms, protozoans, taxonomic richness, tropical bromeliads

## Abstract

Ecological communities hosted within phytotelmata (plant compartments filled with water) provide an excellent opportunity to test ecological theory and to advance our understanding of how local and global environmental changes affect ecosystems. However, insights from bromeliad phytotelmata communities are currently limited by scarce accounts of microfauna assemblages, even though these assemblages are critical in transferring, recycling, and releasing nutrients in these model ecosystems. Here, we analyzed natural microfaunal communities in leaf compartments of 43 bromeliads to identify the key environmental filters underlying their community structures. We found that microfaunal community richness and abundance were negatively related to canopy openness and vertical height above the ground. These associations were primarily driven by the composition of amoebae and flagellate assemblages and indicate the importance of bottom‐up control of microfauna in bromeliads. Taxonomic richness of all functional groups followed a unimodal relationship with water temperature, peaking at 23–25°C and declining below and above this relatively narrow thermal range. This suggests that relatively small changes in water temperature under expected future climate warming may alter taxonomic richness and ecological structure of these communities. Our findings improve the understanding of this unstudied but crucial component of bromeliad ecosystems and reveal important environmental filters that likely contribute to overall bromeliad community structure and function.

## 1 Introduction

Aquatic communities occupying container habitats in plants (phytotelmata) have been used as a model system for testing fundamental ecological theory (Kitching, 2001, 2004; Srivastava et al., 2004). Tank bromeliad species (family: Bromeliaceae) are widely distributed, locally abundant and house‐rich aquatic biota (Cascante‐Marin et al., 2006; Gentry & Dodson, 1987). This allows highly replicated natural experiments across a broad geographical range and analyses of generality of the observed patterns. Recent studies in tank bromeliads have, for instance, advanced our understanding of issues such as top‐down control across a habitat‐size gradient (Petermann, Farjalla, et al., 2015), relative consumption of autochthonous and allochthonous resources in aquatic food webs (Farjalla et al., 2016), or the community consequences of global change in rainfall and temperature regimes (Marino et al., 2017; Pires, Marino, Srivastava, & Farjalla, 2016; Romero, Piccoli, de Omena, & Goncalves‐Souza, 2016). However, the large majority of these advances come from studies focused on a targeted subset of these diverse communities—aquatic macroinvertebrates from both the water and detritus within phytotelmata. Although protozoan and metazoan microfauna assemblages are a critical component of bromeliad food webs (Carrias, Cussac, & Corbara, 2001; Srivastava & Bell, 2009), they have received relatively little attention and remain poorly understood.

Diverse assemblages of aquatic microfauna (composed of flagellates, ciliates, amoebae, rotifers, copepods, oligochaetes, nematodes, flatworms) are important consumers of bacteria and microalgae and serve as prey for larger invertebrate consumers. The intermediate position of microfauna in these ecological networks plays a pivotal role in the transfer, recycling, and release of nutrients (Laessle, 1961; Sherr & Sherr, 1988). Microfauna can be particularly important in the rosettes of tank bromeliads with high detritus content as a main resource for aquatic invertebrates (Brouard et al., 2012). However, there is no comprehensive analysis of factors governing the structure of bromeliad microfaunal communities, also precluding our full understanding of the energy and nutrient transfers in these microhabitats (Marino et al., 2017).

Ecological communities are assembled from the regional species pool by three key processes: biotic filtering, dispersal, and environmental sorting (Chase, 2003; Srivastava & Kratina, 2013). We have previously manipulated homogenized microfaunal communities in Costa Rican tank bromeliads to exclude priority effects and tested whether these communities assemble through top‐down forces, competition for resources or dispersal limitation (Petermann, Kratina, et al., 2015). We found no effects of dispersal (see also Farjalla et al., 2012) and weak top‐down control of mosquito larvae on community assembly. Our analysis showed that the bottom‐up effect of detrital resources is the main driver of experimental microfauna community structure, at least in the short term. This work also indicated that canopy openness and water temperature can impose some constraints on which taxa persist in each particular habitat (Petermann, Kratina, et al., 2015), prompting a comprehensive test of environmental sorting in naturally assembled microfaunal communities.

Previous accounts linking environmental conditions to bromeliad microfauna community structure are sparse. The few studies that have been conducted suggest that light and bromeliad volume are important. For example, open habitats with bromeliads exposed to more light and with more bacteria often have higher microalgal biomass than bromeliads located under closed canopy (Brouard et al., 2011; Laessle, 1961). Rotifers are also positively associated with the total incident radiation, but negatively associated with the height of bromeliads above the ground (Brouard et al., 2012). In French Guiana, protozoan richness increases with bromeliad water volume and their densities were positively associated with rotifer and macroinvertebrate densities (Carrias et al., 2001). A contrasting pattern is found in the lowlands of Panama, with lower densities of rotifers and nematodes recorded in larger as compared to smaller bromeliads (Zotz & Traunspurger, 2016). These results highlight the fact that taxonomic richness and relative densities of individual functional groups can differentially respond to environmental factors and indicate that these responses can be governed by local food web interactions (Srivastava & Bell, 2009).

Here, we conducted a survey of 309 natural microfaunal communities in leaf compartments of 43 bromeliads to assess which environmental mechanisms control community structure and richness patterns of this important but understudied food web component. Based on previous research, we hypothesized that canopy openness, volume of the water (habitat size), and temperature are the main structuring forces, but there will be differential responses to environment of individual functional groups. Such comprehensive and systematic analysis of bromeliad microfauna and their environmental drivers has not been performed previously. This study together with our experimental manipulations (Petermann, Kratina, et al., 2015) thus provides a solid foundation for establishing a link between the macroinvertebrate food webs and the microfaunal food webs inhabiting bromeliads.

## 2 Materials and methods

### 2.1 Study area and data collection

This study was conducted near the Estación Biológica Pitilla in the Area de Conservación Guanacaste, northwestern Costa Rica (10°59′N, 85°26′W). We surveyed 43 large bromeliads of genus *Werauhia* (formerly *Vriesea,* Bromeliaceae) in a 0.5‐km^2^ area at an altitude of approximately 700 m. The habitat the bromeliads were found in is comprised of primary and secondary tropical forests and horse pastures, providing a range of environmental conditions. We extracted microfaunal communities from 27 large bromeliads evenly distributed across environmental conditions and habitat sites. These bromeliads were later used for an experimental manipulation (Petermann, Kratina, et al., 2015). We also extracted microfaunal communities from an additional 16 bromeliads, to include all large bromeliads in the vicinity of the field station. Three to nine samples were taken from each bromeliad, from the phytotelmata at bottom, middle, and top central positions of the plants. The field sampling was carried out within ten days in April and May 2010, at the beginning of the rain season.

We characterized key environmental and structural variables hypothesized to affect microfaunal communities. Prior to sampling, we used portable meters to measure in situ dissolved oxygen (DO), water temperature (°C), and pH (Analion PM608). We characterized canopy openness above the center of each bromeliad plant, using a 35‐mm‐lens camera and calculating the proportion of visible sky in digital images by counting pixels. To quantify detrital resources, we extracted all leaf litter submerged in individual phytotelmata, dried in a propane oven for 40 min, and weighted to the nearest gram. Using silicon tubes, we extracted and measured the natural water content (ml) from all plants. To evaluate microhabitats, we measured the bromeliad size (i.e., diameter in cm) as the maximum distance between the tips of the leaves, number of live bromeliad leaves, and the height of each bromeliad above ground (0–2.5 m). Water volume represents a good approximation of the habitat size, whereas bromeliad diameter and the number of leaves forming wells describe microhabitat structure for the inhabiting communities (Petermann, Farjalla, et al., 2015).

We collected 1 ml water samples with microfaunal communities that were fixed with Lugol's iodine solution (5%) and shipped to University of British Columbia (Vancouver, Canada) for identification. Organisms were identified to “morphotaxa” and counted under an inverted microscope (200× magnification) using and extending a photographic key developed by Thomas Bell during an earlier study at the same location (Srivastava & Bell, 2009). We used a dissecting microscope (Leica) to identify the main groups in 50 μl subsamples placed on dissecting slides. It is important to consider our richness and abundance data as relative, because some species can only be distinguished in live samples. The data collection was carried out under research permit N° ACG‐PI‐028‐2010 (Ministerio del Ambiente, Energía y Telecomunicaciones).

### 2.2 Statistical analyses

We used linear mixed effects (LME) models to identify the impact of multiple environmental variables on estimated microfaunal abundance and richness (richness refers to the total number of species per community, or alpha diversity). We then classified all taxa into five major functional groups (microalgae, flagellates, ciliates, predators, and amoebae) and carried out LME analyses for each group. Environmental conditions, including canopy openness above the plants, subsurface water temperature, pH, amount of leaf litter (detritus), water volume, elevation above ground (vertical height), bromeliad size, number of live bromeliad leaves, were treated as fixed independent variables. We treated the individual bromeliads as a random factor and accounted for the position of phytotelmata within bromeliads, which sorter identified the samples, and species abundances (for the taxonomic richness analysis) as covariates (Pinheiro & Bates, 2000). This conservative approach removes zero values of the abundance covariate from the subsequent analysis. Species abundances were log‐transformed prior to the analyses to achieve normality and improve homoscedasticity of residuals. The relationship between water temperature and microfaunal richness indicated unimodal, rather than linear, relationship. For this reason, we also fit the model with quadratic (polynomial) terms for temperature, accounting for the sorter effect and using individual bromeliads as a random factor. We then compared the models with linear and quadratic (polynomial) terms for temperature using a maximum likelihood ratio test.

To assess which environmental variables alter the microfaunal community composition, we used redundancy analysis (RDA; Legendre and Legendre 1998. RDA is a commonly used form of linear ordination that directly relates multiple taxonomic compositions to several measured environmental factors (direct gradient analysis). We pooled the species within each functional group and then performed the RDA on a Hellinger‐transformed functional group abundances (i.e., dividing the abundance of each functional group in a sample by the total abundance of functional groups of that sample, and taking the square root of that value) in order to reduce the influence of outliers (Legendre and Gallagher 2001). We aggregated individual communities (phytotelmata) within bromeliad plants into lower, intermediate, and upper positions, with the upper position being closest to the central reservoir of the plant, and accounted for position of the community within bromeliad and for the effect of sorter identity. Significance of each environmental variable was determined using Monte Carlo permutation tests (999 permutations) on the results of the RDA. The responses of individual groups (microalgae, flagellates, ciliates, predators, amoebae) to different environmental variables can be visualized in the redundancy ordination plot by overlaying species positions with environmental vectors. All statistical analyses were performed in R 3.3.1 (R Development Core Team, 2016), using R‐packages *nlme* and *vegan*.

## 3 Results

### 3.1 Taxonomic richness

**Figure 1:**
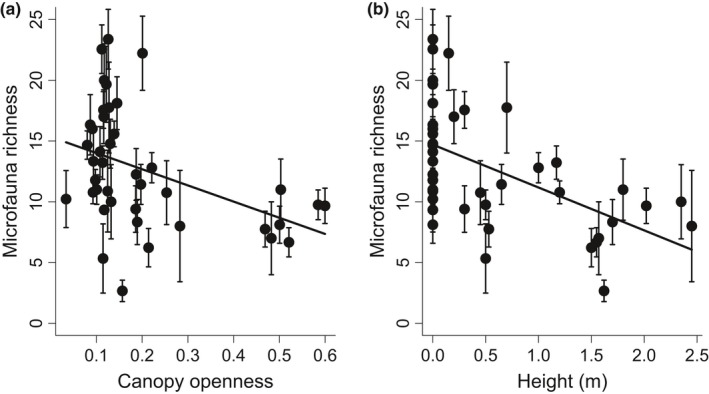
Mean taxonomic richness (number of species) of microfauna community in bromeliads declines with (a) canopy openness (measured as a proportion of visible sky, where 1 represents completely open and 0 means completely closed canopy) and with (b) vertical height of bromeliads above the ground. Data points represent mean values for each bromeliad ± 1 standard error

We detected 109 taxa of microfauna in all bromeliads, and there were 13.40 ± 0.44 (mean ± SE) taxa per sample. After accounting for the effect of sorter identity, position within bromeliad, and log abundance of all microfauna, we found that estimated richness declined with canopy openness (*p* < .002, LME, Figure 1a) and with height above the ground (*p* = .019, LME, Figure 1b). Changing the order of environmental variables in the model did not have any effect on the outcome of the analyses, indicating that collinearity between environmental predictors is not biasing our results.

**Figure 2:**
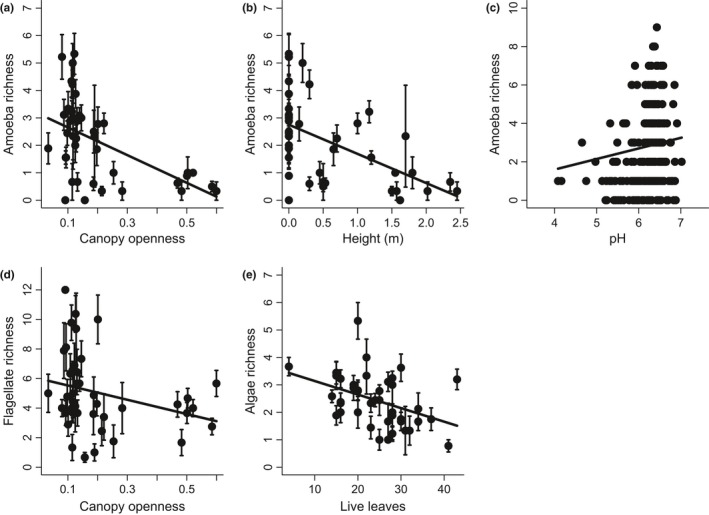
Environmental and structural (number of leaves) conditions that had the strongest effects on taxonomic richness (number of species) of individual functional groups. Canopy openness was measured as a proportion of visible sky above each bromeliad (where 1 represents completely open and 0 means completely closed canopy). Data points in (a), (b), (d), and (e) represent mean values for each bromeliad ± 1 standard error. Measurements from all phytotelmata are shown in panel (c)

To better understand these environmental effects, we then focused on the individual functional groups of microfauna. Amoebae were negatively affected by canopy openness (*p* = .044, LME, Figure 2a) and vertical height above the ground (*p* = .013, LME, Figure 2b). This functional group was also positively associated with pH (*p* = .003, LME, Figure 2c). Canopy openness had a marginal negative effect on richness of flagellates (*p* = .056, LME, Figure 2d). In addition to the trend of the mean along the canopy openness, we also observed the decreased variance in flagellate richness and thus this relationship should be considered with caution. The overall pattern between microfauna richness and canopy openness (Figure 1a) was not influenced by the remaining three functional groups. The number of live bromeliad leaves had positive effect on richness of microalgae (*p* = .026, LME, Figure 2e).

**Figure 3:**
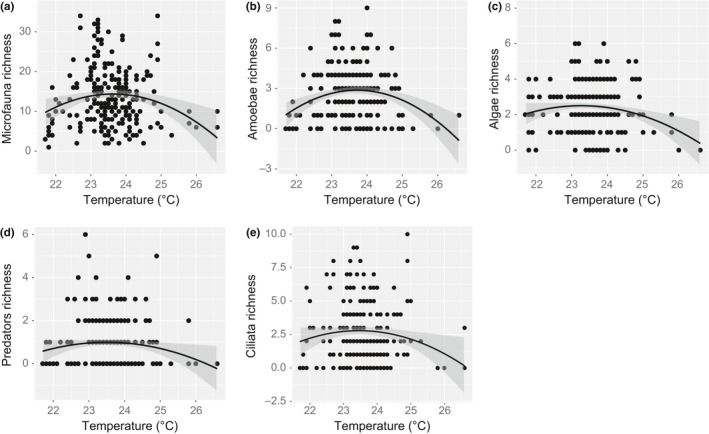
Unimodal relationships between environmental temperature and (a) microfauna taxonomic richness (*p* = .0197), (b) amoeba richness (*p* = .044), (c) microalgal richness (*p* = .029), (d) predatory microfauna richness (*p* = .0432), and (e) ciliate richness (*p* = .0118). Black lines represent quadratic linear model fits, and the gray‐shaded areas are ±95% confidence intervals. The predatory microfauna include rotifers, copepods, oligochaetes, nematodes, and flatworms

Microfauna richness first increased, reached a peak, and then declined across the gradient of water temperature (Figure 3a). The unimodal relationship (polynomial regression) fitted the data significantly better than the linear relationship (*p* = .0197, maximum likelihood model comparison, Figure 3a). Unimodal relationships were also detected for amoebae (*p* = .044, Figure 3b), microalgae (*p* = .029, Figure 3c), predatory microfauna (*p* = .0432, Figure 3d), and ciliates (*p* = .0118, Figure 3e), with the quadratic term performing significantly better in each case. In contrast, taxonomic richness of flagellates and microfauna abundance were not related to temperature (*p* = .1454 and *p* = .7202, respectively, maximum likelihood model comparison).

### 3.2 Microfauna abundance and community composition

**Figure 4:**
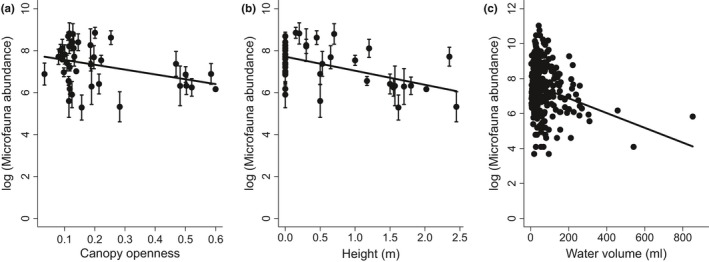
Mean log‐transformed microfauna abundance in bromeliads declines with (a) canopy openness (measured as a proportion of visible sky, where 1 represents completely open and 0 means completely closed canopy), with (b) height of bromeliads above the ground, and with (c) water volume. Data points in (a) and (b) represent mean values for each bromeliad ± 1 standard error, and measurements from all phytotelmata are shown in panel (c)

**Figure 5:**
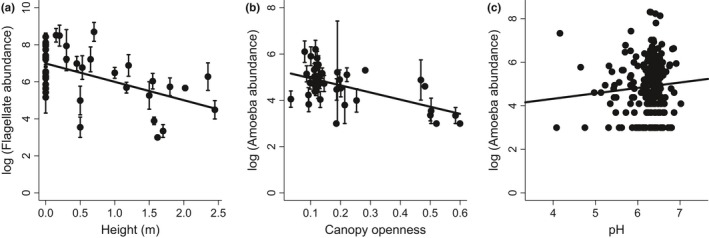
Environmental and structural conditions that had the strongest effects on abundance of individual functional groups. Canopy openness was measured as a proportion of visible sky above each bromeliad (where 1 represents completely open and 0 means completely closed canopy). Data points in (a) and (b) represent mean values for each bromeliad ± 1 standard error. Measurements from all phytotelmata are shown in panel (c)

We found a mean microfauna abundance of 4,436.69 ± 438.60 individuals (mean ± SE) per sample. Estimated microfaunal abundance was reduced by canopy openness (*p* = .017, LME, Figure 4a), height above the ground (*p* = .004, LME, Figure 4b), and water volume (*p* = .017, LME, Figure 4c). However, canopy openness had no significant effect when placed as a last variable in the model, suggesting that it occurred in models largely through collinearity with other environmental variables. Flagellates and amoebas were two functional groups whose abundance significantly responded to environmental conditions. Flagellate abundances were negatively associated with height above the ground (*p* = .003, LME, Figure 5a). Amoeba abundances were negatively associated with canopy openness (*p* < .001, LME, Figure 5b), but positively associated with water pH (*p* = .0153, LME, Figure 5c).

**Figure 6:**
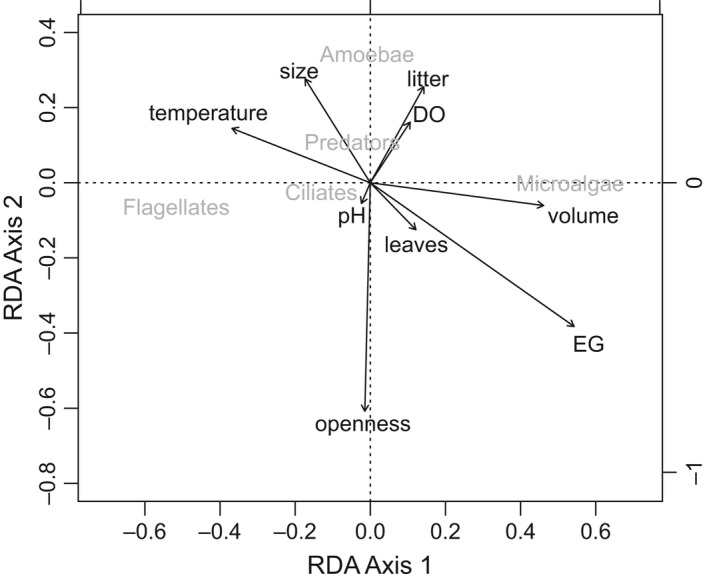
Redundancy analysis (RDA) showing the effect of environmental variables (black) on the Hellinger‐transformed abundances of the main microfauna groups (dark gray) after accounting for the sorter effect and position of individual communities within bromeliads. Canopy openness (proportion of visible sky above each bromeliad), water volume, elevation above ground (EG), and temperature (the longest vectors) were the four variables explaining a significant proportion of the functional group variance (adj. r^2^: .135, *p* < .05). DO represents dissolved oxygen in the water

Microfaunal community composition was largely driven by four environmental variables: canopy openness (*p* = .008, F = 4.772, RDA), height above ground (*p* = .005, F = 5.299, RDA), water volume (*p* = .017, F = 4.120, RDA), and water temperature (*p* = .014, F = 4.319, RDA). According to the RDA, amoebae were negatively associated with canopy openness, and both amoebae and flagellates were negatively associated with height above ground and water volume (Figure 6). Microalgae were positively associated with height above ground and water volume, but negatively associated with water temperature (Figure 6). When forward selection RDA was used, only height above ground and canopy openness remained significant (*p* = .005). Ciliates and predatory microfauna were clustered close to the RDA centroid (Figure 6).

## 4 Discussion

This study indicates that environmental filtering is critical to understanding the local differences among bromeliads in microfauna community structure. Canopy openness and height above ground were identified as the two main factors governing the diversity, abundance, and relative composition of individual functional groups. Canopy openness is a complex variable that integrates multiple direct and indirect effects on natural communities. Higher canopy cover indicates more detritus and throughfall, thus increasing the resource concentrations available to microfauna. In contrast, lower canopy cover results in increased light incidence above the bromeliads and a shift from detrital‐based to more microalgal‐ and rotifer‐dominated communities, favoring autochthonous primary production (Brouard et al., 2011, 2012; Farjalla et al., 2016; Laessle, 1961). Open bromeliad habitats have also been shown to have greater temporal fluctuations in temperature, dissolved oxygen, and CO_2_ (Laessle, 1961; Neutzling, 2015). More stable conditions in bromeliads growing in the shaded habitats may thus support richer and more abundant microfaunal communities.

Bromeliads positioned on the ground tend to have on average more basal resources due to the higher detritus concentration and throughfall, and are likely exposed to the lower incident radiation than epiphytic bromeliads. Furthermore, different rates and modes of dispersal likely contribute to the composition of communities at different heights above ground (Maguire, 1963; Vanschoenwinkel, et al., 2008). Whereas the exact mechanism underlying the negative relationship between the height above ground and microfauna richness and abundance is unknown and likely comprises multiple factors, vertical position on the host tree, light incidence above bromeliads, and particulate organic matter were also three major factors driving the relative abundances of several microfauna groups in French Guyana (Brouard et al., 2012).

Amoebae and flagellates responded the most strongly to the environmental conditions. Flagellates were the most abundant group in our study. This group includes taxa with very short generation times (Laybourn‐Parry, 1992) that are known to respond quickly to environmental change (Walker, Kaufman, & Merritt, 2010). While amoebae are often assumed to respond more slowly (Wallace & Merritt, 1980), their similarly strong response to changing conditions indicates a strong role of environmental sorting and possibly adaptations to the specific set of conditions. Similar to other studies (Laessle, 1961), we found relatively acidic environment in bromeliad phytotelmata (pH 4–7) although amoebae seem to prefer more neutral pH conditions (Figures 2c and 5c). This suggests that amoebal abundances are depressed by ambient pH in most bromeliads.

Water temperature is another important environmental filter for many species and across all ecosystems (Dell, Pawar, & Savage, 2011). The relationship between temperature and richness of all taxonomic groups, except of flagellates, exhibited a unimodal pattern, peaking at 23–25°C. Previous studies proposed that tropical ectotherms in relatively equitable environments have narrower physiological thermal tolerances (Woodward, Perkins, & Brown, 2010) and occupy habitats relatively closer to their thermal limits than their counterparts at higher latitudes (Deutsch et al., 2008; Huey et al., 2009). Our results indicate that a small increase in temperature, and potentially increased temperature variation, could push thermally sensitive taxa out of their tolerance limits and reduce richness of local microfaunal communities. However, the unimodal relationships were contingent on a relatively low number of studied communities (n = 6) at higher temperatures, urging further investigations.

Microcosms studies are usually used as the first empirical tests of novel ecological and evolutionary theory that can combine high power (replication) with complex experimental designs, often impossible to achieve in the field (Altermatt et al., 2015; Gülzow, Muijsers, Ptacnik, & Hillebrand, 2016; Kratina, Hammill, & Anholt, 2010). Natural microcosms, such as those of bromeliads, often include high diversity of invertebrates and are exposed to environmental variation, thus representing a useful transition between models, laboratory systems, and large‐scale natural ecosystems (Kitching, 2004; Srivastava et al., 2004). Our study calls for the integration of microfauna into the ecological and evolutionary research conducted in natural bromeliad microcosms and highlights the importance of environmental sorting. Canopy openness and height above ground are both complex factors, aggregating multiple direct and indirect impacts on the bromeliad microecosystems. Although the effect of detritus concentration itself was not significant in our study, there is now an emerging pattern of a strong bottom‐up forcing (Petermann, Kratina, et al., 2015) and potential control of environmental stability on the microfaunal communities. Nutritional quality of detritus and availability of specific carbon compounds are the key factors defining bacterial community composition (Felip, Pace, & Cole, 1996; Kominoski, Hoellein, Kelly, & Pringle, 2009)—the main resource for many microfaunal groups. Consequently, both the concentration and composition of detritus should be considered if we are to fully understand regulation of microfauna and macrofauna communities in natural ecosystems. Finally, our results also suggest the sensitivity of many functional groups to temperature and contribute to advancing our understanding of the impact of environmental change on ecosystem structure and function.

## Conflict of interest

None declared.
